# Predicting life-threatening hemoptysis in traumatic pulmonary parenchymal injury using computed tomography semi-automated lung volume quantification

**DOI:** 10.1186/s13244-024-01849-8

**Published:** 2024-11-15

**Authors:** Wen-Ruei Tang, Chao-Chun Chang, Chen-Yu Wu, Chih-Jung Wang, Tsung-Han Yang, Kuo-Shu Hung, Yi-Sheng Liu, Chia-Ying Lin, Yi-Ting Yen

**Affiliations:** 1 Division of Thoracic Surgery, Department of Surgery, National Cheng Kung University Hospital, College of Medicine, National Cheng Kung University, Tainan, Taiwan; 2 Division of Trauma and Acute Care Surgery, Department of Surgery, National Cheng Kung University Hospital, Tainan, Taiwan; 3 Department of Medical Imaging, National Cheng Kung University Hospital, College of Medicine, National Cheng Kung University, Tainan, Taiwan

**Keywords:** Computed tomography, Hemoptysis, Pulmonary contusion, Quantitative CT analysis, Thoracic trauma

## Abstract

**Objectives:**

Chest computed tomography (CT) can diagnose and assess the severity of pulmonary contusions. However, in cases of severe lung contusion, the total lung volume ratio may not accurately predict severity. This study investigated the association between life-threatening hemoptysis and chest CT imaging data on arrival at the emergency department in patients with pulmonary contusions or lacerations due to blunt chest injury.

**Methods:**

The records of 277 patients with lung contusions or lacerations treated at a trauma center between 2018 and 2022 were retrospectively reviewed. The ratio of the local lung contusion volume to lobe volume in each lobe was calculated from chest CT images. The maximal ratio in the Hounsfield unit (HU) range was defined as the highest ratio value within the HU range among five lobes.

**Results:**

The median patient age was 41 years, and 68.6% were male. Life-threatening hemoptysis occurred in 39 patients. The area under the receiver operating characteristic curve for the maximal ratio at −500 HU to 100 HU was 96.52%. The cutoff value was 45.49%. Multivariate analysis showed a high maximal chest CT ratio ≥ 45.49% at −500 HU to 100 HU (adjusted odds ratio [aOR]: 104.66, 95% confidence interval [CI]: 21.81–502.16, *p* < 0.001), hemopneumothorax (aOR: 5.18, 95% CI: 1.25–21.47, *p* = 0.023), and chest abbreviated injury scale (AIS, aOR: 5.58, 95% CI: 1.68–18.57, *p* = 0.005) were associated with life-threatening hemoptysis.

**Conclusions:**

Maximal chest CT ratios ≥ 45.49% at −500 HU to 100 HU, hemopneumothorax, and high chest AIS scores are associated with life-threatening hemoptysis in patients with blunt chest trauma.

**Critical relevance statement:**

The present study provides an objective index derived from chest CT images to predict the occurrence of life-threatening hemoptysis. This information helps screen high-risk patients in need of more intensive monitoring for early intervention to improve outcomes.

**Key Points:**

- Emergency department CT helps predict life-threatening hemoptysis in patients with lung contusions.
- Maximal CT ratios ≥ 45.49% (−500 HU to 100 HU, either lung lobe) are associated with life-threatening hemoptysis.
- High chest abbreviated injury scale scores and hemopneumothorax also predict life-threatening hemoptysis.

**Graphical Abstract:**

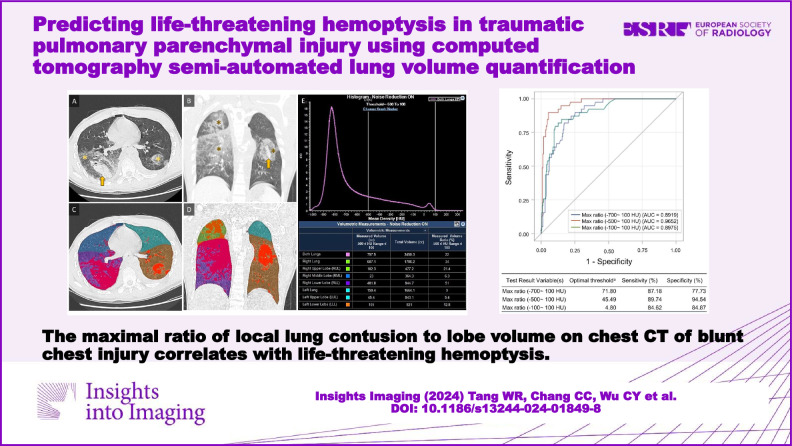

## Introduction

Pulmonary contusion is a common lung injury caused by blunt chest trauma, with an incidence of approximately 50% [1–3] and a mortality rate of 20% [4]. Patients with severe chest trauma may develop pulmonary contusions or parenchymal lacerations, leading to life-threatening hemoptysis; in most cases, urgent lung resection is necessary to control bleeding [5]. Therefore, a fast, convenient, and reliable examination method to establish the increased risk of life-threatening hemoptysis would be useful and may improve outcomes.

Chest computed tomography (CT) images provide fast and quantifiable information to detect pulmonary contusions immediately after injury and have become a standard imaging examination for patients with chest trauma. Studies using quantitative CT analysis have shown that a ratio of affected lung volume to total lung volume ≥ 20% in patients with pulmonary contusions in the acute stage is associated with worse in-hospital outcomes, especially developing complications such as acute respiratory distress syndrome (ARDS) or pneumonia [6–10].

Given that severe lung contusions or lacerations in one of the five lobes may lead to life-threatening hemoptysis, the ratio derived from the total lung volume may not represent the injury severity or the risk of hemoptysis. We hypothesized that the ratio of the contusion volume to the individual lobe volume would better predict the risk of life-threatening hemoptysis in patients with chest trauma. Therefore, this retrospective observational study aimed to investigate the association between life-threatening hemoptysis and chest CT imaging data obtained upon arrival at the emergency department (ED) in patients with pulmonary contusions or lacerations due to blunt chest trauma. Intense monitoring of patients at a high risk of life-threatening hemoptysis may lead to earlier interventions and improved outcomes.

## Methods

**Fig. 1:**
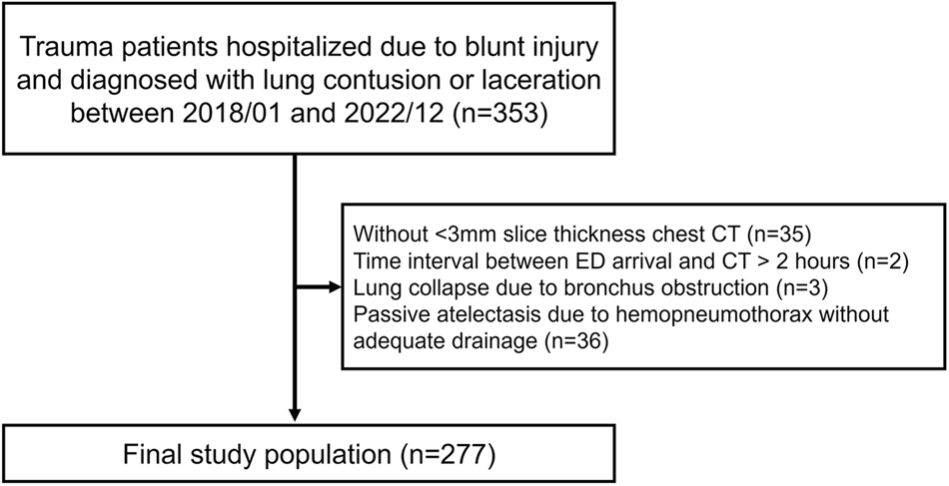
Flowchart of patient selection

Records of patients with blunt chest trauma treated at a level I trauma center between 2018 and 2022 were retrospectively reviewed (Fig. 1). The inclusion criteria were as follows: (1) blunt traumatic chest injury and (2) a diagnosis of lung contusion or laceration. Exclusion criteria were as follows: (1) lack of chest CT with a slice thickness of < 3 mm; (2) interval between arrival at the ED and CT imaging longer than 2 h; (3) lung collapse due to bronchial obstruction; and (4) passive atelectasis due to hemopneumothorax without adequate drainage, defined as a distance between the lung margin and the chest wall at the level of the hilum of > 2 cm [11]. The study protocol was reviewed and approved by the Institutional Review Board, and the requirement for signed informed consent was waived owing to the retrospective nature of the study.

Demographic information and clinical data on arrival at the ED, including the Glasgow coma score (GCS), blood pressure (BP), heart rate, and SpO_2_, were collected. The trauma severity was evaluated by the abbreviated injury scale (AIS) [12, 13] and the injury severity score (ISS) [14]. Patients’ conditions at CT imaging were also recorded. Intubation was defined as an endotracheal tube during CT images. Inspiration depth was evaluated by semi-quantitatively accessing the posterior part of the trachea 2 cm above the carina. Expiration was defined as inwards placement of the posterior part, and inspiration was defined as flat or outwards placement [15–17].

The in-hospital outcomes included life-threatening hemoptysis, length of hospital stay (LOS), intensive care unit (ICU) stay, survival, lung resection, and venous-arterial extracorporeal membrane oxygenation (VA-ECMO) use. Life-threatening hemoptysis was defined as hemoptysis of > 100 mL in 24 h that caused abnormal gas exchange, airway obstruction, or hemodynamic instability [18].

### CT protocol and quantitative image analysis

Unenhanced CT images were acquired using Siemens SOMATOM Definition Flash, Siemens SOMATOM Definition AS, SOMATOM Definition Edge (Siemens Health Ltd., Erlangen, Germany), and GE Optima CT660 (GE Healthcare, USA) CT systems. Imaging was performed with patients in the supine position with full inspiration. The CT parameters were 120 kVp, a tube current of 150–200 mA with automatic tube current modulation, a section thickness of 0.625–1.5 mm, and an image size of 512 × 512 pixels.

**Fig. 2:**
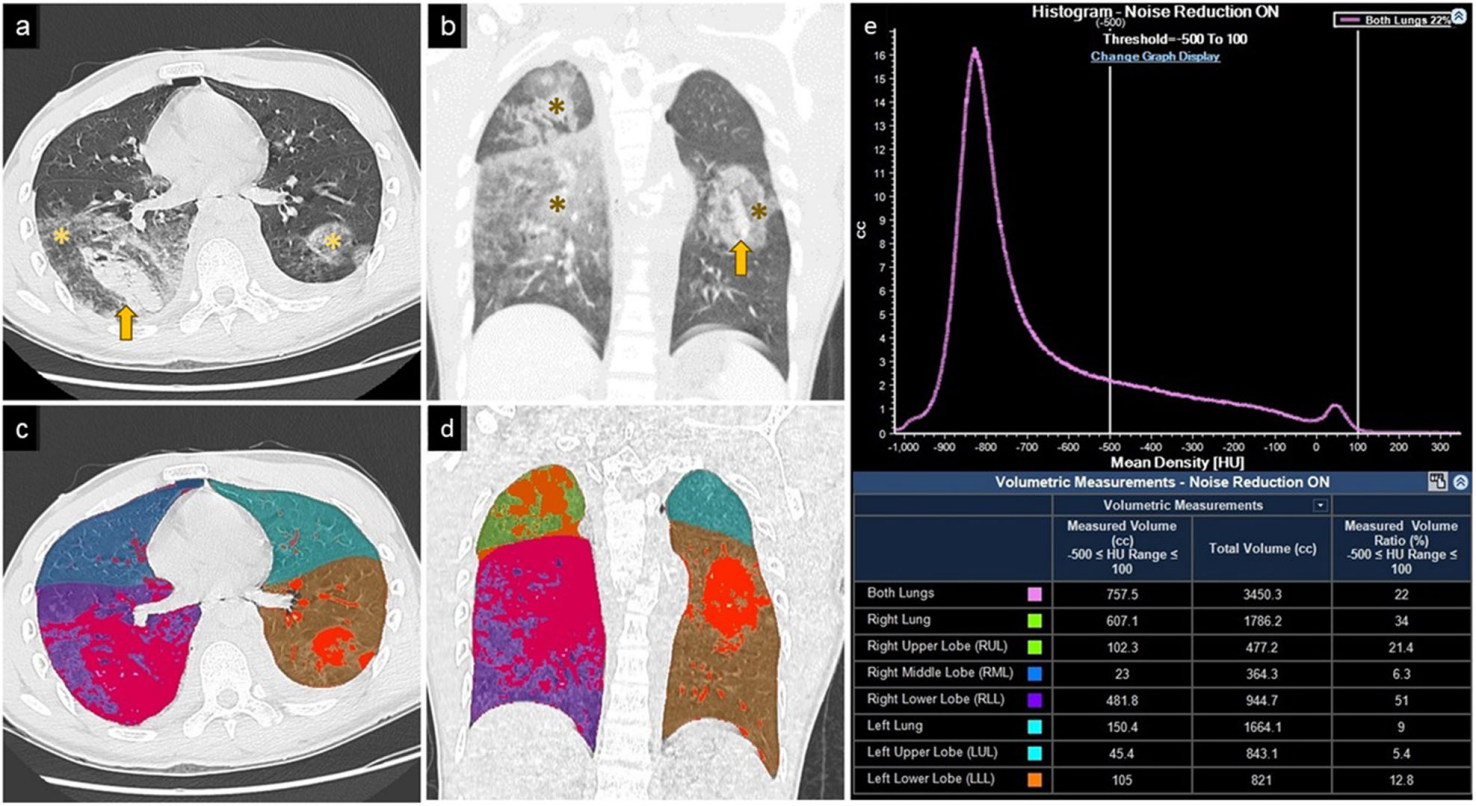
Lung injury in a 22-year-old man following a motor vehicle accident. The patient was hemodynamically stable on arrival with a GCS score of 15, and oxygen saturation of 100% under a simple oxygen mask 8 L/min usage. **a** Axial and (**b**) coronal non-enhanced chest CT images show lung contusion (asterisk) and laceration (arrow). The red voxel represents density (%) between HU = −500  and +100 in corresponding (**c**) axial and (**d**) coronal color map images. **e** Corresponding histogram of whole lung density and pulmonary lobar volumetry. The measured volume ratios (%) between HU = −500 and +100 in each lobe were RUL = 21.4%, RML = 6.3%, RLL = 51%, LUL = 5.4%, and LLL = 12.8%. After two hours the patient developed life-threatening hemoptysis and underwent emergent thoracotomy and RLL lobectomy. GCS, Glasgow coma score; CT, computed tomography; HU, Hounsfield unit; RUL, right upper lobe; RML, right middle lobe; RLL, right lower lobe; LUL, left upper lobe; LLL, left lower lobe

All CT images were quantitatively analyzed using validated lung analysis software (IntelliSpace Portal, Philips Healthcare). The software analyzed whole three-dimensional (3D) CT volumes and presented a probability map showing the voxels belonging to each lobe. The window setting was 1600/−650 HU (*W*/*L*). Sharp convolution B60f kernels were used. The quantitative image analysis steps described by Ippolito et al [19] were used with modifications. Briefly, the border of each lung lobe was identified, and the tracheobronchial tree and proximal vasculature were automatically removed using the software. Each lobe was segmented to allow delineation using an automated lobe segmentation algorithm [20]. The operator evaluated whether automated lobe segmentation was appropriate. According to Gattinoni et al [21], a well-aerated lung volume was defined as a quantity of lung tissue ≤ −700 Hounsfield units (HU) on inspiratory CT; a poorly inflated lung volume was defined as a quantity of lung tissue between −500 HU and −100 HU on inspiratory CT; and a non-inflated lung volume was defined as a quantity of lung tissue between −100 HU and 100 HU on inspiratory CT. The percentage of the local lung contusion volume relative to the lobe volume in each lobe was calculated using the software. The maximal ratio in a given HU range was defined as the highest ratio value within the areas of the given HU range among the five lobes (Fig. 2).

### Statistical analysis

Continuous data are presented as the median and interquartile range (IQR) because of a non-normal distribution and were analyzed using the Wilcoxon rank-sum test. Categorical data are presented as counts and percentages and were analyzed using the chi-square or Fisher’s exact test, as appropriate. Receiver operating characteristic (ROC) curve analysis was conducted to determine the optimal threshold for the maximal ratio of CT results for different lung densities and life-threatening hemoptysis. The area under the ROC curve (AUC) was calculated to determine the model accuracy. The optimal threshold was defined as the cutoff point on the ROC curve corresponding to the Youden index: (sensitivity + specificity) −1. The lung density area was categorized based on three ranges: −700 HU to 100 HU, −500 HU to 100 HU, and −100 HU to 100 HU. The maximal ratio was defined as the maximal value in the five lung lobes within the three threshold ranges. The effect of the maximal ratio (according to the optimal threshold) on life-threatening hemoptysis was calculated using multiple logistic regression and presented as adjusted odds ratios (aOR) and 95% confidence intervals (CI). Adjusted covariates with *p*-values < 0.1 in the comparisons of life-threatening hemoptysis were included in the multivariate analysis. All statistical analyses were performed using SAS statistical software (version 9.4; Cary, NC, USA).

## Results

This study included 277 patients, and their characteristics are shown in Table 1. The median patient age was 41.0 years, the median weight was 65.0 kg, and 68.6% of the patients were male. Most injuries were caused by traffic accidents (83.4%). The following median values were recorded upon admission to the ED: GCS score, 13; systolic BP, 130 mmHg; diastolic BP, 80 mmHg; heart rate, 92 beats/min; and SpO_2_, 97%. The median ISS score was 22.0, and the chest AIS had the highest median value compared to the other AIS values (3.0 vs 0–2.0).

**Table 1:** Patient characteristics

Characteristic	All patients, (*n* = 277)
Age, years	41.0 (22.0–63.0)
Male sex, (%)	190 (68.6)
Body weight, (kg)	65.0 (54.5–75.0)
Mechanism of injury	
Assault	2 (0.7)
Fall	44 (15.9)
Traffic accident	231 (83.4)
Initial vital signs in ED	
GCS score	15.0 (11.0–15.0)
Systolic BP, (mm Hg)^a^	130.0 (114.0–148.0)
Diastolic BP, (mm Hg)^a^	80.0 (70.0–93.0)
Heart rate, (beat/min)^a^	92.0 (81.0–107.0)
SpO_2_, (%)^a^	97.0 (94.0–100.0)
Pneumothorax only	47 (17.0)
Hemothorax only	42 (15.2)
Hemopneumothorax	82 (29.6)
Intubation	68 (24.6)
Inspiration	255 (92.1)
Trauma severity	
ISS score	22.0 (14.0–33.0)
Head and neck AIS	2.0 (0.0–3.0)
Face AIS	0.0 (0.0–1.0)
Chest AIS	3.0 (3.0–3.0)
Abdomen AIS	0.0 (0.0–3.0)
Extremity AIS	2.0 (0.0–3.0)
External AIS	0.0 (0.0–0.0)
CT results	
Maximal ratio (−700 < HU < 100)	60.5 (39.3–76.5)
Maximal ratio (−500 < HU < 100)	23.6 (14.4–40.4)
Maximal ratio (−100 < HU < 100)	1.3 (0.5–4.7)
In-hospital outcomes	
Life-threatening hemoptysis	39 (14.1)
LOS, (days)	10.0 (5.0–19.0)
ICU stay, (days)	1.0 (0.0–7.0)
Survival	248 (89.5)
Lung resection	31 (11.2)
VA-ECMO	5 (1.8)

Data are presented as median (IQR), or count (percentage)

*ED* emergency department, *GCS* Glasgow coma scale, *BP* blood pressure, *ISS* injury severity score, *AIS* abbreviated injury scale, *CT* computed tomography, *HU* Hounsfield unit, *LOS* length of hospital stay, *ICU* intensive care unit, *VA-ECMO* venous-arterial extracorporeal membrane oxygenation, *OHCA* out-of-hospital cardiac arrest

^a^ Incomplete data because nine patients due to OHCA when arrived at the hospital

The median maximal ratio determined by CT was 60.5% for −700 HU to 100 HU, 23.6% for −500 HU to 100 HU, and 1.3% for −100 HU to 100 HU. Of 277 patients, 14.1% had life-threatening hemoptysis, 89.5% survived, 11.2% underwent lung resection, and 1.8% were treated with VA-ECMO.

**Fig. 3:**
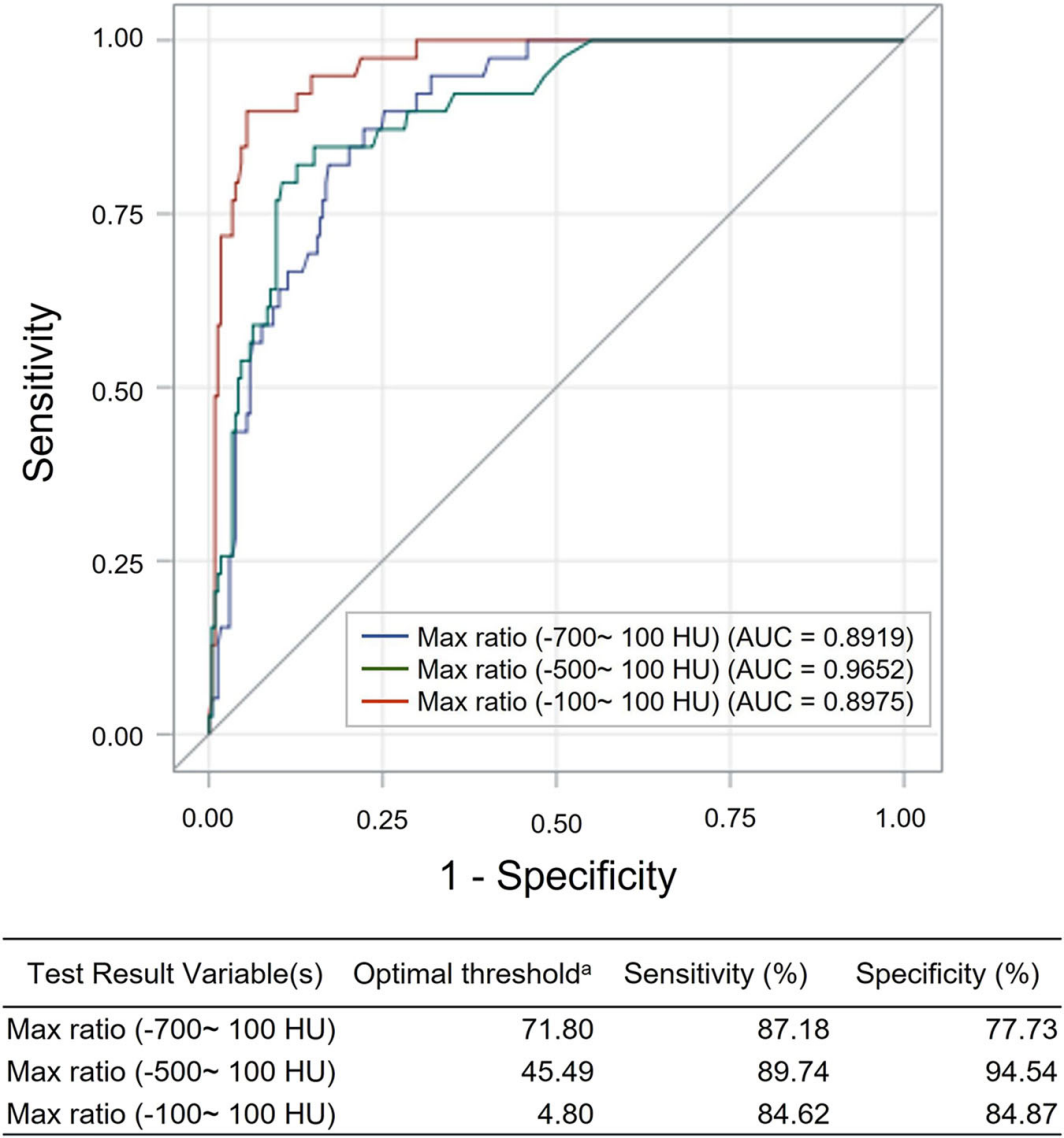
ROC curve for life-threatening hemoptysis in different lung density ranges. ^a^The optimal threshold is the cutoff point on the corresponding ROC curve with Youden’s Index: (sensitivity + specificity) − 1

The ROC curves for life-threatening hemoptysis and different lung density areas are shown in Fig. 3. The AUC of the maximal lung density area ratios for −700 HU to 100 HU, −500 HU to 100 HU, and −100 HU to 100 HU were 89.19%, 96.5%, and 89.75%, respectively. The maximal ratio of the −500 HU to 100 HU lung density area had the highest accuracy for predicting life-threatening hemoptysis (*p* < 0.001, compared with −700 HU to 100 HU, Table 2). The optimal threshold was 45.49, the sensitivity was 89.74%, and the specificity was 94.54%.

**Table 2:** Comparison of AUC between different lung density ranges

Test result variable, (s)	AUC (%) (95% CI)	Diff of AUC (%) (95% CI)	*p*-value
Max ratio (−700 HU to 100 HU)	89.19 (84.84–93.54)	Ref	
Max ratio (−500 HU to 100 HU)	96.52 (94.19–98.85)	**7.33 (3.83–10.83)**	**<** **0.001**
Max ratio (−100 HU to 100 HU)	89.75 (84.87–94.63)	0.56 (− 5.53 to 6.66)	0.857

Significant results are shown in bold

*AUC* area under the ROC curve, *CI* confidence interval, *HU* Hounsfield unit

A comparison between a high maximal ratio (≥ 45.49%) and a low maximal ratio (< 45.49%) within −500 HU to 100 HU is shown in Table 3. Compared to patients with a low maximal ratio, patients with a high maximal ratio were heavier (median: 70.0 kg vs 64.4 kg, *p* = 0.041), had a lower GCS score (10.0 vs 15.0, *p* < 0.001), a lower SpO_2_ (93.5% vs 98.0%, *p* = 0.007), higher frequencies of hemopneumothorax (69.2% vs 23.1%, *p* < 0.001) and intubation (50% vs 19.2%, *p* < 0.001), a lower frequency of inspiration (83.3% vs 93.9%, *p* = 0.034), a higher ISS score (35.0 vs 22.0, *p* < 0.001), higher head and neck AIS (2.0 (0.0–4.0) vs 2.0 (0.0–3.0), *p* = 0.018), higher chest AIS (4.0 vs 3.0, *p* < 0.001), and higher abdominal AIS (2.0 vs 0, *p* = 0.047). The frequencies of life-threatening hemoptysis, lung resection, VA-ECMO use, longer ICU stay, and lower survival were significantly higher in patients with a higher maximal ratio than in those with a lower maximal ratio (all *p* < 0.001).

**Table 3:** Comparison between high (≥ 45.49) and low maximal ratio (< 45.49) of −500 HU to 100 HU

	High, (*n* = 48)	Low, (*n* = 229)	*p*-value
Age	37.5 (22.0–60.0)	42.0 (23.0–64.0)	0.561
Sex, (male)	38 (79.2)	152 (66.4)	0.083
Body weight	70.0 (60.0–77.5)	64.4 (52.8–75.0)	**0.041**
Mechanism of injury			0.580
Assault	0 (0.0)	2 (0.9)	
Fall	10 (20.8)	34 (14.8)	
Traffic accident	38 (79.2)	193 (84.3)	
Initial vital signs in ED			
GCS score	10.0 (3.0–15.0)	15.0 (13.0–15.0)	**<** **0.001**
Systolic BP, (mmHg)	128.0 (107.0–148.0)	130.5 (114.0–148.0)	0.694
Diastolic BP, (mmHg)	80.0 (69.0–109.0)	80.0 (70.0–92.0)	0.838
Heart rate, (beat/min)	94.0 (81.0–122.0)	91.5 (81.0–104.0)	0.238
SpO_2_, (%)	93.5 (88.0–100.0)	98.0 (95.0–100.0)	**0.007**
Pneumothorax only	3 (7.7)	44 (18.5)	0.096
Hemothorax only	3 (7.7)	39 (16.4)	0.161
Hemopneumothorax	27 (69.2)	55 (23.1)	**<** **0.001**
Intubation	24 (50.0)	44 (19.2)	**<** **0.001**
Inspiration	40 (83.3)	215 (93.9)	**0.034**
Trauma severity			
ISS score	35.0 (23.0–48.0)	22.0 (13.0–29.0)	**<** **0.001**
Head and neck AIS	2.0 (0.0–4.0)	2.0 (0.0–3.0)	**0.018**
Face AIS	0.0 (0.0–1.0)	0.0 (0.0–1.0)	0.764
Chest AIS	4.0 (3.0–4.0)	3.0 (3.0–3.0)	**<** **0.001**
Abdomen AIS	2.0 (0.0–3.5)	0.0 (0.0–3.0)	**0.047**
Extremity AIS	2.0 (0.0–3.0)	2.0 (0.0–3.0)	0.861
External AIS	0.0 (0.0–0.0)	0.0 (0.0–0.0)	0.901
In-hospital outcome			
Life-threatening hemoptysis	35 (72.9)	4 (1.7)	**<** **0.001**
Hospitalization, (day)	14.0 (4.5–23.5)	9.0 (5.0–17.0)	0.310
ICU stay, (day)	6.5 (1.0–14.5)	0.0 (0.0–5.0)	**<** **0.001**
Survival	32 (66.7)	216 (94.3)	**<** **0.001**
Lung resection	26 (54.2)	5 (2.2)	**<** **0.001**
VA-ECMO	5 (10.4)	0 (0.0)	**<** **0.001**

Significant results were shown in bold

*ED* emergency department, *GCS* Glasgow coma scale, *BP* blood pressure, *ISS* injury severity score, *AIS* abbreviated injury scale, *CT* computed tomography, *HU* Hounsfield unit, *LOS* length of hospital stay, *ICU* intensive care unit, *VA-ECMO* venous-arterial extracorporeal membrane oxygenation

The results of the univariate and multivariate analyses of life-threatening hemoptysis and a maximal ratio within −500 HU to 100 HU are shown in Table 4. Patients with life-threatening hemoptysis had a significantly higher frequency of a high maximal ratio (89.7% vs 5.5%, *p* < 0.001), lower GCS score (8.0 vs 15.0, *p* < 0.001), lower SpO_2_ (93.0% vs 98.0%, *p* = 0.001), higher frequencies of hemopneumothorax (69.2% vs 23.1%, *p* < 0.001) and intubation (56.4% vs 19.3%, *p* < 0.001), lower frequency of inspiration (82.1% vs 93.7%, *p* = 0.022), higher ISS score (36.0 vs 22.0, *p* < 0.001), higher head and neck AIS (2.0 (0.0–5.0) vs 2.0 (0.0–3.0), *p* = 0.005), and higher chest AIS (4.0 vs 3.0, *p* < 0.001). After adjusting for hemopneumothorax, Intubation, inspiration, GCS score, ISS score, head and neck AIS, and chest AIS, the high maximal ratio group had a 105-fold greater risk of life-threatening hemoptysis compared to the low maximal ratio group (model 1: aOR = 104.66, 95% CI: 21.81–502.16, *p* < 0.001). High chest AIS (aOR = 5.58, 95% CI: 1.68–18.57, *p* = 0.005) and hemopneumothorax (aOR = 5.18, 95% CI: 1.25–21.47, *p* = 0.023) were also associated with life-threatening hemoptysis. After heart rate and SpO_2_ were added to model 1 (model 2), the high maximal ratio group had a 118-fold greater risk of life-threatening hemoptysis (aOR = 117.77, 95% CI: 18.65–743.87, *p* < 0.001). High chest AIS (aOR = 7.52, 95% CI: 1.70–33.25, *p* = 0.008) remained associated with life-threatening hemoptysis.

**Table 4:** Univariate and multivariate analysis of the association between maximal ratio at −500 HU to 100 HU and life-threatening hemoptysis

	Life-threatening hemoptysis			Model 1		Model 2^a^	
	Yes, (*n* = 39)	No, (*n* = 238)	*p-*value	OR (95% CI)	*p-*value	OR (95% CI)	*p-*value
Max ratio (high vs low)	35 (89.7)	13 (5.5)	**<** **0.001**	104.66 (21.81–502.16)	**<** **0.001**	117.77 (18.65–743.87)	**<** **0.001**
Age	37.0 (22.0–59.0)	43.5 (23.0–64.0)	0.210				
Sex (male vs female)	31 (79.5)	159 (66.8)	0.114				
Body weight	68.0 (55.0–72.8)	65.0 (53.1–75.0)	0.683				
Mechanism of injury			0.436				
Assault	0 (0.0)	2 (0.8)					
Fall	9 (23.1)	35 (14.7)					
Traffic accident	30 (76.9)	201 (84.5)					
Initial vital signs in ED							
GCS score	8.0 (3.0–15.0)	15.0 (13.0–15.0)	**<** **0.001**	0.88 (0.71–1.11)	0.290	0.89 (0.68–1.18)	0.425
Systolic BP	128.0 (107.5–146.0)	131.0 (114.0–149.0)	0.462	–			
Diastolic BP	80.0 (68.0–106.5)	80.0 (70.0–92.0)	0.811	–			
Heart rate	99.5 (83.5–125.0)	91.0 (81.0–104.0)	0.059	–		0.99 (0.96–1.03)	0.691
SpO_2_	93.0 (86.0–98.0)	98.0 (95.0–100.0)	**0.001**	–		1.07 (0.95–1.2)	0.266
Pneumothorax only	3 (7.7)	44 (18.5)	0.096				
Hemothorax only	3 (7.7)	39 (16.4)	0.161				
Hemopneumothorax	27 (69.2)	55 (23.1)	**<** **0.001**	5.18 (1.25–21.47)	**0.023**	4.84 (1–23.52)	0.051
Intubation	22 (56.4)	46 (19.3)	**<** **0.001**	1.97 (0.24–16.06)	0.526	1.94 (0.18–21.07)	0.587
Inspiration	32 (82.1)	223 (93.7)	**0.022**	0.66 (0.09–4.71)	0.676	0.56 (0.07–4.49)	0.587
Trauma severity							
ISS score	36.0 (24.0–50.0)	22.0 (13.0–29.0)	**<** **0.001**	1.02 (0.94–1.1)	0.605	1.02 (0.94–1.12)	0.591
Head and neck AIS	2.0 (0.0–5.0)	2.0 (0.0–3.0)	**0.005**	0.83 (0.47–1.44)	0.502	0.76 (0.42–1.4)	0.380
Face AIS	0.0 (0.0–1.0)	0.0 (0.0–1.0)	0.703				
Chest AIS	4.0 (4.0–4.0)	3.0 (3.0–3.0)	**<** **0.001**	5.58 (1.68–18.57)	**0.005**	7.52 (1.70–33.25)	**0.008**
Abdomen AIS	2.0 (0.0–4.0)	0.0 (0.0–3.0)	0.138				
Extremity AIS	2.0 (0.0–3.0)	2.0 (0.0–3.0)	0.411				
External AIS	0.0 (0.0–0.0)	0.0 (0.0–0.0)	0.788				

Adjusted by results with *p*-value < 0.1 in univariate analysis

Significant results are shown in bold

*ED* emergency department, *GCS* Glasgow coma scale, *BP* blood pressure, *ISS* injury severity score, *AIS* abbreviated injury scale, *CT* computed tomography, *HU* Hounsfield unit, *LOS* length of hospital stay, *ICU* intensive care unit, *VA-ECMO* venous-arterial extracorporeal membrane oxygenation

^a^ Nineteen observations were deleted due to missing values for the explanatory variables

The association between a ratio ≥ 45.49% within −500 HU to 100 HU in each lobe and life-threatening hemoptysis is shown in Table 5. After adjusting for related factors, the risk of at least one lung lobe with a ratio ≥ 45.49% leading to life-threatening hemoptysis was 25 times higher than that of all lobes with ratios < 45.49% (model 1, aOR = 25.33, 95% CI: 6.84–93.90). The risk remained 17-fold greater (model 2: aOR = 17.06, 95% CI: 3.95–73.76, *p* < 0.001) after heart rate and SpO_2_ were added to model 1.

**Table 5:** The association between a ratio ≥ 45.49% at − 500 HU to 100 HU in each lobe and life-threatening hemoptysis in multivariate analysis

Lobe with ratio ≥ 45.49%	Life-threatening hemoptysis			Model 1		Model 2^a^	
	Yes, (*n* = 39)	No, (*n* = 238)	*p*-value	aOR (95% CI)	*p*-value	aOR (95% CI)	*p*-value
Numbers							
≥ 1	23 (59.0)	9 (3.8)	**<** **0.001**	25.33 (6.84–93.90)	**<** **0.001**	17.06 (3.95–73.76)	**<** **0.001**
Location							
RUL	11 (28.2)	2 (0.8)	**<** **0.001**				
RML	3 (7.7)	1 (0.4)	**0.009**				
RLL	19 (48.7)	7 (2.9)	**<** **0.001**				
LUL	10 (25.6)	1 (0.4)	**<** **0.001**				
LLL	19 (48.7)	8 (3.4)	**<** **0.001**				

Adjusted by results with *p*-value < 0.1 in univariate analysis in Table 4

Significant results are shown in bold

^a^ Nineteen observations were deleted due to missing values for the explanatory variables

## Discussion

The present study showed that the maximal ratio of the local lung contusion volume to the individual lobe volume on chest CT images of patients with blunt chest injury was associated with life-threatening hemoptysis. Patients with a maximal ratio ≥ 45.49%, as determined using chest CT images at the threshold of −500 HU to 100 HU, have a more than 100-fold greater risk of hemoptysis than patients with a maximal ratio < 45.49%. Hemopneumothorax and a higher chest AIS are also associated with life-threatening hemoptysis. The results provide objective information for screening patients at a high risk of life-threatening hemoptysis, and intense monitoring is needed for early intervention to improve outcomes.

Our results showed that patients with a chest CT maximal ratio ≥ 45.49% at −500 HU to 100 HU have a greatly increased risk of life-threatening hemoptysis, providing objective information for predicting life-threatening hemoptysis. Other studies have reported that quantitative CT analysis is useful for identifying and quantifying pulmonary contusions after blunt chest trauma. A previous study used a CT volume index score based on the affected and total lung volumes to predict the outcomes of polytrauma patients with pulmonary contusions [8]. The results showed that a pulmonary contusion volume of > 20% of the total lung volume was associated with longer total hospital and ICU stays, more days on a ventilator, and the development of pneumonia. Mahmood et al [6] studied 226 patients with blunt chest trauma and reported that a contusion volume of > 20% was associated with ARDS, blood transfusion, and prolonged mechanical ventilation. Other studies also reported that a pulmonary contusion volume of ≥ 20% was significantly associated with an increased risk of ARDS and pneumonia compared to a volume of < 20% [7, 9, 10].

Pulmonary contusion, or damage to the lung parenchyma, is a common injury caused by blunt chest trauma and may be accompanied by pneumothorax, hemothorax, or bronchial injury [5, 22, 23]. However, assessing the degree of lung injury and subsequent risk of complications such as hemothorax and hemoptysis can be difficult. Historically, the severity of lung contusions and the extent of lung parenchymal injury have been evaluated by visual observation of radiographs or CT images by an experienced radiologist. However, this method tends to overestimate or underestimate the amount of damaged lung tissue. In our analysis, we used the ratio of the local lung contusion volume to the individual lobe volume, which provides a more accurate estimate of the severity of lung damage because life-threatening hemoptysis can occur when one of five lobes has severe lung contusions or lacerations. If only a single lobe has a severe contusion, urgent resection should be performed to control bleeding. If the lesion area is too large (≥ 2 lobes), surgery may not be suitable; the option is to suspend treatment or use ECMO for conservative treatment, but the mortality rate is high. Additionally, there is no standard HU range for quantitative CT image analysis to determine the extent of lung contusions. Our analysis adopted the threshold range reported by Gattinoni et al [21] and showed that a range of −500 HU to 100 HU provides the best imaging results for determining the amount of lung tissue affected.

Our results showed that hemopneumothorax and a high chest AIS are associated with life-threatening hemoptysis. Injury severity and hemopneumothorax have been reported to be associated with poor clinical outcomes in patients with blunt chest trauma [24]. Beshay et al [25] reported that chest AIS is an independent risk factor for mortality in patients with thoracic trauma. Bayer et al [26] reviewed the records of 22,000 patients with severe injury in the TraumaRegister DGU^®^ registry and reported that the proportions of chest tube intubation, systolic BP < 90 mmHg, use of catecholamines and cardiopulmonary resuscitation, and needs of massive transfusion and emergency surgery during early hospital care increase with increasing chest AIS. Together with our findings, a higher chest AIS indicates more severe lung lesions at admission; therefore, intense monitoring and aggressive intervention during early clinical management are needed to improve outcomes.

In most situations without the need to manually adjust the range of lobe segmentation, it takes about 90 seconds from image reading to result in a calculation. Unless lobe segmentation is manually adjusted, the same CT image will produce the same results through software calculation. This avoids intra- and inter-rater variability. For CT images of very serious or very mild chest injuries, the inter-rater judgment has high consistency. However, for intermediate chest injuries (e.g., −500 HU to 100 HU ~ 50%), physicians with different experiences may have different points of view. Underestimating the severity may delay the timing for intervention, but aggressive judgment may let the patient receive unnecessary lung resection. The results of the present study can help reduce poor outcomes caused by differences in inter-rater judgment.

### Limitations

This study had several limitations. First, not all patients with pulmonary contusions were included. Patients with major trauma and unstable vital signs were sent directly to emergency surgery without an imaging examination, and some patients with mild symptoms may have undergone only X-rays and not CT imaging or outpatient follow-up without hospitalization; therefore, these patients were excluded from the study. Nevertheless, the study aimed to identify patients who required early intervention. Therefore, failure to include the patients mentioned above should not have affected the results; patients with immediate life-threatening conditions may have already undergone surgery, and those with mild symptoms only required conservative treatment and observation. Second, not all the CT images of patients with blunt chest trauma were applicable to this study. Hemopneumothorax is common in patients with blunt chest trauma. However, severe hemopneumothorax causes lobar collapse, which affects the calculation of lung volume and density. Therefore, CT images of patients with obvious hemopneumothorax but without chest tube drainage or those with tracheobronchial disruption were not applicable to this study.

## Conclusion

Chest CT images with a maximal ratio of affected lung volume to individual lung volume of ≥ 45.49% at a density range of −500 HU to 100 HU, hemopneumothorax, and a high chest AIS are associated with life-threatening hemoptysis in patients with blunt chest trauma. This information helps screen patients with more serious injuries and the need for more intensive monitoring so that early intervention can be introduced to improve outcomes.

## Data Availability

All data analyzed during this study are included in this published article.

## Abbreviations

AIS: Abbreviated injury scale
aOR: Adjusted odds ratio
ARDS: Acute respiratory distress syndrome
AUC: Area under the ROC curve
BP: Blood pressure
CI: Confidence interval
CT: Computed tomography
ED: Emergency department
GCS: Glasgow coma score
HU: Hounsfield unit
ICU: Intensive care unit
IQR: Interquartile range
ISS: Injury severity score
LLL: Left lower lobe
LOS: Length of hospital stay
LUL: Left upper lobe
RLL: Right lower lobe
RML: Right middle lobe
ROC: Receiver operating characteristic
RUL: Right upper lobe
VA-ECMO: Venous-arterial extracorporeal membrane oxygenation
